# Exploitation of Hetero- and Phototrophic Metabolic Modules for Redox-Intensive Whole-Cell Biocatalysis

**DOI:** 10.3389/fbioe.2022.855715

**Published:** 2022-04-13

**Authors:** Eleni Theodosiou, Adrian Tüllinghoff, Jörg Toepel, Bruno Bühler

**Affiliations:** ^1^ Institute of Applied Biosciences, Centre for Research and Technology Hellas, Thessaloniki, Greece; ^2^ Department of Solar Materials, Helmholtz Centre for Environmental Research GmbH—UFZ, Leipzig, Germany

**Keywords:** whole-cell redox biocatalysis, central metabolism, TCA cycle, metabolic engineering, cyanobacteria, redox balance

## Abstract

The successful realization of a sustainable manufacturing bioprocess and the maximization of its production potential and capacity are the main concerns of a bioprocess engineer. A main step towards this endeavor is the development of an efficient biocatalyst. Isolated enzyme(s), microbial cells, or (immobilized) formulations thereof can serve as biocatalysts. Living cells feature, beside active enzymes, metabolic modules that can be exploited to support energy-dependent and multi-step enzyme-catalyzed reactions. Metabolism can sustainably supply necessary cofactors or cosubstrates at the expense of readily available and cheap resources, rendering external addition of costly cosubstrates unnecessary. However, for the development of an efficient whole-cell biocatalyst, in depth comprehension of metabolic modules and their interconnection with cell growth, maintenance, and product formation is indispensable. In order to maximize the flux through biosynthetic reactions and pathways to an industrially relevant product and respective key performance indices (i.e., titer, yield, and productivity), existing metabolic modules can be redesigned and/or novel artificial ones established. This review focuses on whole-cell bioconversions that are coupled to heterotrophic or phototrophic metabolism and discusses metabolic engineering efforts aiming at 1) increasing regeneration and supply of redox equivalents, such as NAD(P/H), 2) blocking competing fluxes, and 3) increasing the availability of metabolites serving as (co)substrates of desired biosynthetic routes.

## 1 Introduction

Living microbial cells have at their disposal active enzymes and metabolic pathways that allow the catalysis of synthetically interesting reactions in an economically feasible way and, in many cases, the simultaneous *in situ* regeneration of required cofactors or cosubstrates. They can theoretically produce all the metabolites present in their metabolic network from simple carbon sources such as sugars, organic acids, or even CO_2_. If naturally occurring microorganisms are employed, the production efficiencies typically are rather low (Chae et al., 2017). For the successful development of robust, productive, and efficient whole-cell biocatalysts, whole-cell systems have to be considered in their entire complexity (Lee et al., 2013; Schrewe et al., 2013; Ladkau et al., 2014; Volmer et al., 2015; Kadisch et al., 2017). Cell physiology is intrinsically optimized to secure growth and not the envisaged bioconversion. The maximization of the crucial process performance parameters titer, yield, and productivity thus typically requires the engineering of metabolic machineries *via* the redesign of existing metabolic pathways and/or the introduction of novel/artificial ones (Chae et al., 2017).

The present review focuses on the exploitation and engineering of modules of hetero- and phototrophic metabolism as well as artificial modules for redox-intensive production schemes. In heterotrophs, the central carbon metabolism provides carbon building blocks as well as redox power and energy by making use of simple organic molecules such as sugars and thus constitutes the hub for nearly all catabolic and anabolic processes. It includes the Embden–Meyerhof–Parnas pathway (EMPP), the Entner-Doudoroff pathway (EDP), the pentose phosphate pathway (PPP), as well as the tricarboxylic acid (TCA) cycle (Macnab, 1990), with few individual variations depending on the ecological niche in which the organism lives (Sudarsan et al., 2014). During aerobic growth, a maximum of 24 reducing equivalents (electrons) in the form of 12 molecules of NADH, NADPH, or FADH_2_ is theoretically obtained per molecule of glucose oxidized to CO_2_ (Blank et al., 2010). Their transfer to O_2_ *via* the respiratory electron transport chain then generates the majority of the metabolic energy. In contrast, photoautotrophic metabolism relies on differing resources for carbon, electrons, and energy, i.e., CO_2_, water, and light, respectively. The light reactions of photosynthesis gather electrons from water using light energy (photosynthetic water oxidation), generate a proton gradient *via* the photosynthetic electron transport chain (PETC), and thereby regenerate NADPH and ATP to fuel CO_2_ fixation *via* the Calvin-Benson-Bassham cycle (Lassen et al., 2014a). The latter feeds glycderaldehyde-3-phosphate (G3P) into gluconeogenesis and lower glycolysis, thereby connecting to EMPP, EDP, and PPP and indirectly also the TCA cycle. In both heterotrophs and photoautotrophs, the central carbon metabolism produces biomass precursors (Chen et al., 2016) and is highly connected to redox and energy metabolism (Liao et al., 2016; Wang et al., 2017).

Phototrophic organisms are increasingly attracting attention as hosts for the sustainable production of C-based products, as they can rely on light, CO_2_, and water for growth and product formation. This allows biocatalyst production, regeneration, and operation at minimal economical and ecological costs in overall carbon neutral and land-saving processes. Recent results show the high potential of cyanobacteria, green algae, and other phototrophs to produce biomass (Tsuzuki et al., 2019), biofuels (Arias et al., 2021; Kato et al., 2022), chemicals (Vavitsas et al., 2021; Jaiswal et al., 2022), and high value compounds (Tanvir et al., 2021). Respective advantages and disadvantages are intensely discussed, and new strategies have been developed to overcome limitations in carbon fixation (Batista-Silva et al., 2020). In particular, electron consuming reactions are prone to benefit from electrons derived from photosynthetic water oxidation, with H_2_ formation as a prominent example (Appel et al., 2020; Lupacchini et al., 2021). Maximal electron transfer rates in phototrophs illustrate their high potential to fuel biocatalytic reactions (Grund et al., 2019). Additionally, the photosynthesis product O_2_ can be utilized to support O_2_-dependent reactions (Hoschek et al., 2017; Hoschek et al., 2018; Tüllinghoff et al., 2022), with the potential to overcome O_2_ limitation, a major bottleneck in many processes based on heterotrophs (Baldwin and Woodley, 2006; Ouyang et al., 2018). However, C- (Angermayr et al., 2015) and e^−^-based (Jodlbauer et al., 2021) processes with phototrophic organisms are still limited by several factors, ranging from the lack of advanced molecular biology tools and a poor understanding of the cellar metabolism and its regulation to the lack of bioreactor systems enabling high cell density cultivation (Yu et al., 2013; Till et al., 2020). Generally heterotrophic organisms can still be considered the favored hosts for most production processes due to 1) higher growth rates, 2) a considerably deeper understanding of metabolic and regulatory networks, 3) a more expanded molecular biology tool box, 4) a very active metabolism, i.e., a high glucose consumption rate, and 5) the high cell density cultivation options. These advantages allow elaborate processes like *in vivo* cascades (Ladkau et al., 2014; Ladkau et al., 2016; Woo et al., 2018) and the production of fine and bulk chemicals at industrial scale. However, as stated above, phototrophic organisms have a high potential as hosts for electron demanding reactions, making glucose or other reduced C-compounds for the regeneration of reduction equivalent dispensable. Thus, bioprocesses based on phototrophs profit from low land use demands. Whereas heterotrophic hosts require—beside a low area demand for the bioreactor plants—arable land for the rather area-inefficient production of glucose or other organic substrates, phototrophs show comparably high areal productivity and can also be applied on non-arable land and buildings (Brandenburg et al., 2021; Fernández et al., 2021; Schipper et al., 2021). As long as glucose remains a rather cheap resource, it will be difficult for photobiotechnology to compete; but facing a growing global population with an increasing demand for food, energy, and chemicals, photobiotechnology can be attributed a high future potential making research on respective molecular biology tools, metabolic and regulatory modules, and high cell density cultivation highly valuable.

Since the synthesis of most target products make use of metabolic modules either providing carbon or energy, maximizing respective fluxes is pivotal. This requires knowledge on structure and function of the metabolic network as well as its response upon environmental or genetic perturbations (Jahan et al., 2016; Aslan et al., 2017). In case of redox-dependent production systems, the rate of redox cofactor regeneration is intrinsically linked with the entirety of metabolic modules producing and consuming redox cofactors (Blank et al., 2010) and thus depends on the chosen microbial production system. Whereas heterotrophs rely on the central carbon metabolism with a bias for NADH over NADPH supply, phototrophs involve the light reaction as main module with a bias for NADPH instead of NADH supply, both with respective challenges in redox balancing. In phototrophs, the light reactions often generate more reducing power than their primary metabolism needs, i.e., their metabolism is sink-limited, so that excess reducing power can be exploited for light-driven biosyntheses (Lassen et al., 2014a). Metabolic cofactor regeneration can become limiting, when the target reaction runs at high rates and has to compete with NAD(P)H demands for maintenance and biomass formation. It is obvious that the bioprocess objective to maximize target product formation is not in line with the natural objective of a cell to exploit its redox and energy metabolism for optimized growth and maintenance (Schrewe et al., 2013). To overcome metabolic and cofactor imbalances that impede whole-cell biocatalyst performance, various engineering strategies have been employed, which will be summarized and discussed in this review. These strategies either aim at increasing regeneration and supply of redox equivalents or at blocking competing fluxes. Thereby, heterotrophic, phototrophic, and introduced modules are considered and discussed regarding their potential to fuel whole-cell redox biocatalysis, including strategies to improve them.

### 2 Rerouting Electron Transfer Using Enzymatic Modules

In microbial metabolism, redox cofactors serve as electron carriers providing the cell with the reducing power needed in energy and/or anabolic metabolism. Furthermore, they play critical roles in maintaining intracellular redox homeostasis and are key players for the production of many, fuels, chemicals, and pharmaceuticals *via* redox biocatalysis (Duetz et al., 2001; Zhao and van der Donk, 2003; Park, 2007; Schroer et al., 2009). Their essential role in host metabolism and the complexity and flexibility of their turnover give metabolism-based cofactor regeneration a high potential, but make respective analyses and metabolic engineering towards efficient microbial cell factories a complex task (Blank et al., 2008; Holm et al., 2010; Lee et al., 2013).

**FIGURE 1:**
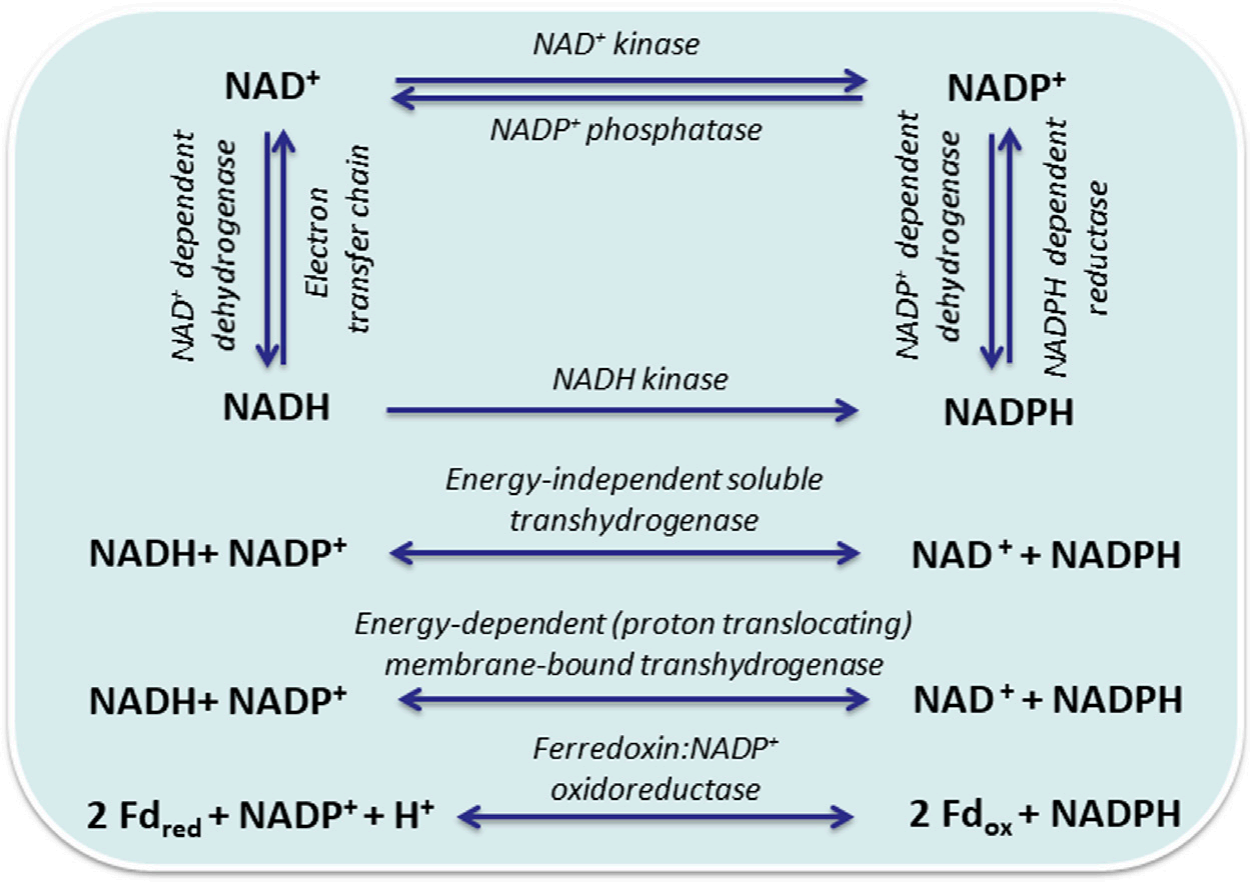
Interconversion and regeneration of redox equivalents via reactions that are not directly coupled to the central carbon metabolism: NAD^+^ kinase (EC 2.7.1.23); NADH kinase (EC 2.7.1.86), NADP^+^ phosphatase (EC 2.7.1.23); NAD^+^-dependent dehydrogenase (EC:1.12.1.2); NADP(H)^+^-dependent dehydrogenases (EC 1.12.1.3); energy-independent soluble pyridine nucleotide transhydrogenase (UdhA, EC 1.6.1.1); energy-dependent proton translocating membrane-bound pyridine nucleotide transhydrogenase (PntAB, EC 1.6.1.2); ferredoxin:NADP^+^ oxidoreductase (FNR, EC 1.18.1.2).

Redox balancing is pivotal for living cells and is affected by any respective manipulation. An important aspect is the different roles, which NAD(H) and NADP(H) play in heterotrophic and phototrophic cells. In the latter, NADPH constitutes a main product of the photosynthetic light reaction, the electron donor for CO_2_ fixation, and thus the central electron carrier under illumination (Ishikawa and Kawai-Yamada, 2019). This changes in the dark, when maintenance metabolism mainly relies on NADH and respiration. In heterotrophs, NADH is the main electron carrier for catabolism and NADPH for anabolism. Both, hetero- and phototrophs can balance pool sizes and reroute electrons between different electron carriers, involving transhydrogenases and NAD kinases (Figure 1). The different roles of transhydrogenases and NAD kinases in hetero- and phototrophs, as well as the usage of heterologous NAD(P)H regeneration systems will be discussed below. Biotechnological approaches based on living cells have to consider not only the electron donor dependency of synthetic reactions and pathways, but also the presence of competing pathways and the cellular responses upon electron drain.

### 2.1 Engineering by Means of NAD(P/H) Transhydrogenases

The function of NAD(P/H) transhydrogenases differs among organisms, especially between phototrophic and heterotrophic bacteria, and is not completely understood, yet. The transhydrogenase reaction (NADH + NADP^+^ ↔ NAD^+^ + NADPH) is catalyzed by the membrane-bound, proton-translocating PntAB (EC 1.6.1.2) or the soluble, energy-independent UdhA (EC 1.6.1.1) (Sauer et al., 2004; Pedersen et al., 2008). Up to now, only Enterobacteriaceae are known to contain both enzymes; all other microbes contain either one or none. Cyanobacteria harbor the genes for PntAB only, which was found to be particularly important for NADPH production under low-light mixotrophic conditions (Kämäräinen et al., 2017). Using mutant *Escherichia coli* strains and metabolic flux analysis (MFA), Sauer et al. (Sauer et al., 2004) identified PntAB as major source of NADPH and reported that it provides 35–45% of the NADPH required during standard aerobic batch growth on glucose, whereas PPP and isocitrate dehydrogenase contributed 35–45% and 20–25%, respectively. On the other hand, hydride transfer from NADPH to NAD^+^ by UdhA was found to be essential for growth under metabolic conditions with excess NADPH formation, such as growth on acetate or with inactivated glucose-6-phosphate isomerase (PGI). Canonaco et al. (2001) demonstrated that, in a Δ*pgi* mutant with the PPP as primary route of glucose catabolism, UdhA activity could efficiently restore the redox balance and improve the growth rate by 25%. They concluded that, in prokaryotes, PntAB catalyzes energy- and NADH-dependent NADP^+^ reduction at low intracellular NADPH levels, whereas UdhA oxidizes NADPH at high intracellular NADPH levels (Sauer et al., 2004).

The function of the transhydrogenases in phototrophic prokaryotes is thought to be similar, maintaining redox homeostasis under imbalanced conditions. Normally, PntAB activity can be neglected during photoautotrophic growth (Kämäräinen et al., 2017). In *Synechocystis* sp. PCC 6803, however, *pntA* transcription was shown to be high under nitrogen and phosphate limitation (Kopf et al., 2014), conditions known to involve an unbalanced NADH/NADPH ratio, suggesting that PntAB plays an important role for redox homeostasis under such conditions. Similarly, it was found that mixotrophic growth, with an enhanced NADH level, depends on PntAB in cyanobacteria (Kämäräinen et al., 2017). A *pntAB*-deficient mutant exhibited growth defects under low-light conditions and day-night rhythm in the presence of glucose. However, the redox state in terms of the NADPH/NADP ratio also appears to be subject of large diurnal variation and to play an important role in respective global regulation (Saha et al., 2016). Further studies on the function of transhydrogenases in phototrophs are missing.

The transhydrogenase systems constitute promising engineering targets to enhance NAD(P)H-dependent biotransformations both in phototrophs and heterotrophs. Because membrane proteins are usually considered more tedious to be heterologously expressed, soluble UdhA is more commonly used in metabolic engineering. Such engineering was successfully employed for the syntheses of several industrially useful compounds. In the study of Xu et al. (2019), a highly NADPH-demanding squalene synthesis pathway was established in *E. coli* to create a squalene-producing bacterial strain. Thereby, *udhA* was overexpressed and obviously enabled an improved intracellular NADPH/NADP^+^ ratio, resulting in a 59% increase in squalene titer. For (S)-2-chloropropionate production from 2-chloroacrylate catalyzed by the NADPH-dependent 2-haloacrylate reductase, Jan et al. (2013) found that the presence of UdhA increases product yield and NADPH availability while the presence of PntAB has the opposite effect. UdhA also conveyed an increase in polyhydroxybutyrate (PHB) productivity and yield in engineered *E. coli* (Sanchez et al., 2006). Coexpression of native *udh*A from *E. coli* together with the *phb* operon from *Alcaligenes eutrophus* H16 from high copy plasmids resulted in an increase in PHB yield from 49 to 66% of total cell dry weight and an increase in final concentration from 3.52 to 6.42 g L^−1^ compared to the control strain expressing only the *phb* operon. These results are somewhat in contrast to those reported by Sauer et al. for redox balancing during growth and indicate that UdhA not only oxidizes NADPH at high NADPH levels, but also contributes to NADPH formation at low NADPH levels caused by a strong NADPH sink (Schroer et al., 2009). Using a thymidine-overproducing *E. coli* strain, the NADPH availability increased by disrupting PGI and overexpressing *nadK* (NAD kinase) or *udhA*. In chemostat cultures, the NADPH/NADP^+^ ratios at steady state correlated positively with thymidine yields (Lee et al., 2010). Fatty acid production also depends on NADPH and has been reported to decrease by 88.8% upon deletion of *pntB* and *udhA* in *E. coli*. Whereas the deactivation of the *zwf* gene decreased production, fatty acid biosynthesis in the *zwf* mutant was recovered upon co‐expression of *pntAB* and *nadK* (Li et al., 2018).

In cyanobacteria, the production of lactic acid is the first example of how the heterologous coexpression of *udhA* (e.g., from *E. coli*, *B. subtilis*) together with lactate dehydrogenase (e.g., from *E. coli, P. aeruginosa*) improves the production due to enhanced NADH supply (Angermayr et al., 2012; Varman et al., 2013). Whereas overexpression of *E. coli udhA* in cyanobacteria improved NADH-dependent production of lactate, phototrophic growth was reduced, probably due to NADPH consumption by UdhA (Niederholtmeyer et al., 2010). Conversely, overexpression of endogenous *pntAB* in *Synechocystis* increased the NADPH-dependent production of 3-hydroxypropionic acid without inhibition of phototrophic growth (Wang et al., 2016). Similarly, *pntAB* overexpression was shown to support NADPH-dependent sorbitol production in *Synechocystis* sp. PCC 6803 (Chin et al., 2018). Also here, a rebalancing of NADPH availability obviously was necessary, enabling, together with an increase in the fructose bisphosphatase level, a nearly 27-fold increase in sorbitol titer compared to the initial strain just overexpressing a sorbitol-6-phosphate dehydrogenase gene.

As mentioned above, transhydrogenases are lacking in some biotechnologically interesting microorganisms. Yamauchi et al. (2014) introduced the *E. coli udhA* and *pntAB* genes into glutamic acid-producing *Corynebacterium glutamicum* and examined the metabolic characteristics of the recombinant strains under aerobic and microaerobic conditions. Introduction of the *udh*A gene did not cause major metabolic changes under both conditions. However, the introduction of *pnt*AB increased the NADH/NAD^+^ ratio under microaerobic conditions and thereby lactic, acetic, and succinic acid formation, pointing towards the potential of PntAB to catalyze a rebalancing towards NADH formation depending on growth conditions. In *Rhodospirillum rubrum*, a purple non-sulfur alphaproteobacterium, the overexpression of *E. coli pntAB* together with *Ralstonia eutropha phaB1*, encoding an NADPH-dependent polyhydroxyalkanoate (PHA)-precursor synthesizing reductase, accumulated poly(3-hydroxybutyrate-co-3-hydroxyvalerate) with a 13-fold higher 3-hydroxyvalerate content compared to the wild type strain (Heinrich et al., 2015). The engineered *R. rubrum* strain was also able to synthesize this industrially relevant copolymer from CO_2_ and CO in syngas. The increased incorporation of 3-hydroxyvalerate was attributed to an excess of the PHA precursor propionyl-CoA, which was generated from aspartate via threonine, thereby consuming two NADPH and producing one NADH, to compensate for PntAB-catalyzed electron transfer from NADH to NADP^+^. The increased 3-hydroxyvalerate incorporation possibly also involved a shortage of the otherwise preferred PHA precursor acetyl-CoA consumed by the NADH generating TCA cycle (Heinrich et al., 2015). Obviously, the function of the two types of transhydrogenases (UdhA and PntAB) is not unidirectional and, depending on the conditions and host organism applied, both can support NADPH or NADH supply.

### 2.2 Overexpression of NAD^+^ kinases

NAD^+^ kinases (NADKs) catalyze the phosphorylation of NAD^+^ to NADP^+^ using ATP as the phosphoryl donor (Kawai et al., 2001). These enzymes are key to balance the ratio of NAD(H) to NADP(H). In an engineered thymidine producing *E. coli* strain, it was reported that *nadK* overexpression resulted in a shift of the NADPH/NADP^+^ ratio from 0.184 to 0.267. However, the overall (NADH + NADP^+^)/(NADPH + NAD^+^) ratio remained constant probably by regulating *pntAB* and *udhA* expression levels to compensate for the effects of *nad*K overexpression (Lee et al., 2009). The *nad*K overexpression in a recombinant *E. coli* harboring a polyhydroxybutyrate (PHB) synthesis pathway doubled the overall PHB accumulation in a bioreactor experiment and came along with a 3- to 6-fold increase in NADP^+^ concentration (Li et al., 2009). The overexpression of the NADK gene *yfjB* together with *pntAB* in *E. coli* was reported to increase NADPH availability and thereby isobutanol production to a larger extent than that of *pntAB* alone, whereas *yfjB* overexpression alone had no effect (Shi et al., 2013). This beneficial synergistic effect was more prominent under anaerobic conditions, when NADH constitutes the main electron carrier, PPP and TCA cycle are usually not functional, and transhydrogenases are the only source for NADPH. In general, NADPH-dependent bioproduction routes of thymidine (Lee et al., 2010), isobutanol (Shi et al., 2013), PHB (Li et al., 2009; Zhang et al., 2015), and shikimic acid (Cui et al., 2014) in *E. coli* and of L-arginine (Rahman et al., 2012) and L-isoleucine (Shi et al., 2012) in *Corynebacterium* species were enhanced via *nadK* overexpression alone or combined with genes encoding other NADPH-related enzymes.

In cyanobacteria, NADKs have several functions. Proteome search for NADK homologs of the 72 species registered in CyanoBase revealed 69 species having two typical NADKs classified as Slr0400 type (group 1) and Sll1415 type (group 2) (Ishikawa and Kawai-Yamada, 2019). It is hypothesized that the two NADK types possess distinct functions in cyanobacteria. Ishikawa et al. (2019) reported that a sll1415-deficient mutant showed a growth-impaired phenotype under conditions that would typically yield photomixotrophy (12-h light/12-h dark cycling), or heterotrophy (light-activated heterotrophic growth, LAHG, with cells in darkness exposed to light for 15 min every 24 h) and that wild type cells exhibited 2.2- and 1.8-fold increased NADP^+^ and NADPH levels, respectively, 96 h after shifting from autotrophic to photomixotrophic conditions. This suggests a key role of group 2 NADKs in photoheterotrophic growth. On the other hand, it is speculated that group 1 NADKs play a key role in suppressing heterotrophic metabolism in *Synechocystis* sp. PCC 6803 under photoautotrophic conditions (Ishikawa and Kawai-Yamada, 2019). A *slr0400*-deficient mutant accumulated NAD^+^, resulting in unsuppressed activity of glycolysis and TCA cycle enzymes (Ishikawa et al., 2021). Further research is necessary to clarify the exact role of the two NADK types in cyanobacteria. Effects of NADK gene overexpression and deletion on target product formation have not been investigated, yet.

### 2.3 Exploiting Heterologous NAD(P)H Generating Systems

**FIGURE 2:**
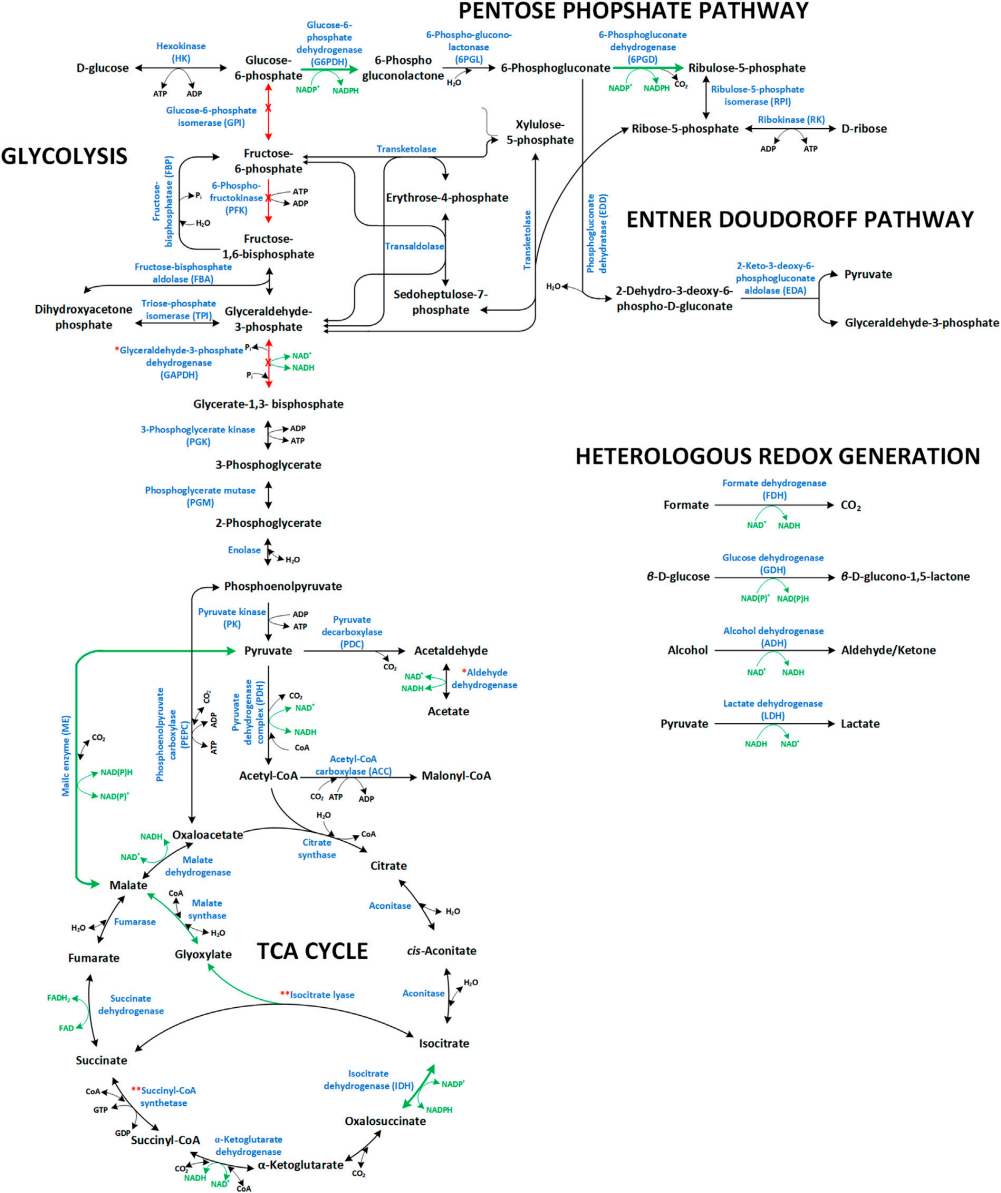
Overview of the NAD(P)^+^/NAD(P)H dependent reactions in central carbon metabolic modules (glycolysis, pentose phosphate pathway, TCA cycle, and Entner-Doudoroff pathway) and heterologous redox cofactor regeneration systems commonly used to fuel redox-intensive production processes. Green arrows and red arrows indicate reactions, which have been upregulated and downregulated/deleted, respectively, to promote/improve redox intensive production schemes. Enzyme names are accompanied by their abbreviations as cited in the text. Respective genes are: the PPP genes *zwf* (glucose 6-phosphate dehydrogenase, **G6PDH**) and *gnd* (6-phosphogluconate dehydrogenase, **6PGD**) both encoding NADPH forming enzymes (Lim et al., 2002; Poulsen et al., 2005; Zhang et al., 2016a); the glycolytic genes *pgi* (glucose-6-phosphate isomerase, **GPI**), *pfkA*/B (phosphofructokinase I/II, **PFK**), and *gap*A (G3P dehydrogenase, **GAPDH**); the TCA cycle genes *maeA/B* (malic enzyme*,* **ME**) and *icd* (Isocitrate dehydrogenase, **IDH**). The symbol (^*^) denotes enzymes that can be exchanged with functionally equivalent isozymes but with different cofactor preference (i.e., NADP^+^-dependent variants of aldehyde dehydrogenase and GAPDH). The symbol (^**^) denotes reactions that have been either activated or deactivated depending on the envisaged redox related metabolic engineering target, as in the case of isocitrate lyase and succinyl-coA synthetase. With activated glyoxylate shunt, more malate is formed which in turn can be converted to pyruvate by the ME involving NADPH formation. In order to promote α-KG dependent redox biotransformations, the glyoxylate shunt has been deactivated forcing metabolic flux through the introduced enzyme (e.g., proline or isoleucine dioxygenases). Succinyl-CoA synthetase activity significantly promotes NADPH allocation (*via* IDH) for NADPH-dependent syntheses (e.g., xylitol production). On the other hand, deletion or reduction of this activity promotes α-KG-dependent redox biotransformations.

Apart from the metabolic engineering of intrinsic NAD(P)H generating pathways, studies have focused on exploiting heterologous systems to increase intracellular NAD(P)H availability and enhance the efficiency and productivity of NAD(P)H-dependent whole-cell biotransformations (Figure 2). Of those, glucose dehydrogenases (GDHs) and formate dehydrogenases (FDHs) are most widely used. The first catalyzes the oxidation of β-D-glucose to β-D-glucono-1,5-lactone with simultaneous reduction of NAD(P)^+^ to NAD(P)H, the latter the NAD^+^-dependent oxidation of formate to CO_2_.

Berríos-Rivera et al. (2002) manipulated the NADH availability in *E. coli* by regenerating NADH through the heterologous expression of *Candida boidinii* FDH regenerating one mole of NADH per mole of formate converted to CO_2_. Natively, this FDH converts formate to CO_2_ and H_2_ without cofactor involvement. Under anaerobic conditions, the NADH availability increased from 2 to 4 mol NADH per mol glucose consumed and promoted ethanol formation. Under aerobic conditions, when formate was not formed by metabolism but added to the medium, the increased NADH availability stimulated usually inactive fermentation pathways, i.e., ethanol, lactate, and succinate formation (Berríos-Rivera et al., 2002). Wang et al. (2013) introduced either GDH from *B. subtilis* or FDH from *C. boidinii* into a 2,3-butanediol (BD) dehydrogenase bearing *E. coli* for the production of (2S, 3S)-2,3-BD from diacetyl, i.e., a liquid fuel and a building block in asymmetric synthesis. The engineering not only improved the efficiency of (2S, 3S)-2,3-BD production, but also simplified product isolation by driving the reaction to completion and preventing the accumulation of gluconic, acetic, and lactic acid that are by-produced when only glucose is used for internal cofactor regeneration. Introduction of FDH resulted in higher (2S, 3S)-2,3-BD concentration, productivity, and yield, coming along with a large increase in the intracellular NADH concentration. However, GDH introduction poorly improved whole-cell biocatalysis efficiency, probably due to poor glucose permeation over cellular membranes and intracellular gluconic acid accumulation. *E. coli* essentially takes up glucose *via* active transport, i.e., the phosphotransferase system (PTS). Thereby, glucose is directly phosphorylated and does not become available for GDH. In crude extracts, GDH showed high activity, whereas the slow glucose permeation into cells limited GDH-mediated NADH regeneration (Wang et al., 2013). Schewe et al. showed that the introduction of the glucose facilitator from *Zymomonas mobilis*, mediating energy-independent glucose diffusion into cells, can overcome this limitation in CYP450 bearing *E. coli* for the NADPH-dependent oxyfunctionalization of α-pinene to α-pinene oxide, verbenol, and myrtenol (Schewe et al., 2008). FDH from *Mycobacterium vaccae* was applied for efficient NADH supply in *E. coli* for D-fructose reduction of to D-mannitol by coexpressed mannitol dehydrogenase from *Leuconostoc pseudomesenteroides* (Kaup et al., 2004)*.* The additional introduction of the *Z. mobilis* glucose facilitator thereby improved D-fructose uptake, enabling 84% yield. Whereas GDH from *B. subtilis* has been extensively used for *in vivo* NAD(P)H regeneration, mainly because of its dual cofactor specificity and ease of expression in *E*. *coli*, interesting biochemical properties (e.g., activity and stability) and cofactor preference have been reported for GDHs from different organisms (Pongtharangkul et al., 2015). Pongtharangkul et al. (2015) cloned and expressed in both *E. coli* and *B. subtilis* a GDH gene from *B. amyloliquefaciens* (GDH-BA) and evaluated the kinetics and biochemical properties of the purified enzyme. They suggested that the high specific activity as well as the low *K*_M_-value towards glucose and NADP^+^ make this GDH a promising candidate for *in vivo* applications. Recombinant *B. subtilis* harboring GDH-BA was successfully used to regenerate NADPH for n-hexane hydroxylation catalyzed by *B. subtilis* containing the CYP450 BM3 variant F87V (Pongtharangkul et al., 2015).

By expressing *adh* from *Lactobacillus brevis* in *E. coli*, Ernst et al. (2005) developed whole-cell biotransformation systems for the reduction of prochiral carbonyl compounds to chiral hydroxy acid derivatives. The *fdh* gene from *M. vaccae* N10 thereby was coexpressed for NADH regeneration and, in the presence of formate, increased the intracellular NADH/NAD^+^ ratio 7-fold. This enabled the formation of 40 mM methyl (R)-3-hydroxy butanoate from methyl acetoacetate with 100% yield. Also *gdh* coexpression together with genes encoding carbonyl reductases in *E. coli* has been shown to be valuable for the production of chiral alcohols (Kataoka et al., 2003).

For the production of chemicals from CO_2_, also the photoautotrophic redox metabolism gains increasing attention. However, the cofactor bias of phototrophs, with NADPH as main source of reduction equivalents, differs from that of heterotrophs. For D-lactate production, Li et al. changed the cofactor preference of the key enzyme D-lactate dehydrogenase (LdhD) from NADH to NADPH. The engineered LdhD in *Synechococcus elongatus* PCC 7942 then enabled efficient light-driven production of optically pure D-lactate from CO_2_. Mutation of LdhD increased D-lactate productivity by over 3.6-fold. The additional introduction of a lactic acid transporter and bubbling CO_2_-enriched air further enhanced D-lactate concentration and productivity (Li et al., 2015a). For phototrophic D-lactate synthesis from CO_2_ by *Synechocystis* sp. PCC 6803, Varman et al. (2013) utilized a mutated GDH. Together with codon optimization and heterologous expression of a soluble transhydrogenase, this approach significantly improved D-lactate synthesis. Whereas *in vivo* NAD(P)H regeneration systems have been widely studied, NAD(P)^+^ regeneration has been less explored and can be considered less demanding. Using the H_2_O producing NADH oxidase from *L. brevis* as heterologous NAD^+^ regenerating enzyme together with a NAD^+^-dependent (2R, 3R)-2,3-BD dehydrogenase from *B. subtilis*, Xiao et al. (2010) developed an *E. coli* biocatalyst for the production of (3S)-acetoin from meso-2,3-BD, (3R)-acetoin from (2R, 3R)-2,3-BD, and (2S, 3S)-2,3-BD from a substrate mixture. Their work demonstrated that coupling NADH oxidase from *L. brevis* with NAD^+^-dependent dehydrogenases provides an efficient platform for NAD^+^-dependent whole-cell biotransformations.

## 3 Pathway Engineering

Central carbon metabolism generally refers to the main NAD(P)H-generating catabolic pathways. Whereas NADH especially fuels aerobic energy metabolism, NADPH is consumed especially in anabolic pathways. In photoautotrophs, NADPH additionally functions as provider of reduction equivalents for CO_2_ fixation and thus constitutes the main carrier of reduction equivalents under light conditions. Heterotrophs rely on catabolism for NAD(P)H regeneration with a bias towards NADH. Catabolic modules have been widely exploited to fuel redox-intensive production processes. Key enzymes involved in catabolic NAD(P)H regeneration are summarized in Table 1, which gives respective reactions, cofactor specificities, and pathway affiliations. In the following Sections 3.1 and 3.2, approaches followed for the engineering of these pathways for NADPH and NADH regeneration are summarized and discussed. Further, the main approaches for heterotrophic metabolism are visualized in Figure 2. Section 3.3 then focusses on the engineering of phototrophic metabolic modules for whole-cell redox biocatalysis. At this point, the value of NAD(P)H biosensors as useful tools to identify genetic manipulations leading to improved NADPH availability is highlighted (Zhang et al., 2016a).

**TABLE 1:** NAD(P)H generating enzymes in central carbon metabolism.

Enzyme	Reaction	Involved pathway
Glucose-6-phosphate dehydrogenase (G6PDH; EC:1.1.1.49)	D-glucose 6-phosphate + NADP^+^ → 6-phospho-D-glucono-1,5-lactone + NADPH + H^+^	PPP, EDP
NAD(P)^+^ glucose dehydrogenase (GDHs; EC:1.1.1.47, EC:1.1.1.119)	D-glucose + NAD(P)^+^ → D-glucono-1,5-lactone + NAD(P)H + H^+^	PPP, EDP
6-Phospho-D-gluconate dehydrogenase (6PGDH; EC:1.1.1.44)	6-phospho-D-gluconate + NADP^+^ → D-ribulose 5-phosphate + CO_2_ + NADPH + H^+^	PPP
NADP^+^-dependent non-phosphorylating glyceraldehyde 3-phosphate dehydrogenase (GAPN; EC:1.2.1.9)	D-glyceraldehyde 3-phosphate + NADP^+^ + H_2_O → 3-phospho-D-glycerate + NADPH + 2 H^+^	EMPP, PPP
NAD(P)^+^-dependent phosphorylating glyceraldehyde 3-phosphate dehydrogenase (NAD(P)^+^-GAPDH; EC:1.2.1.12, 1.2.1.13)	D-glyceraldehyde 3-phosphate + phosphate + NAD(P)^+^ → 3-phospho-D-glyceroyl phosphate + NAD(P)H + H^+^	EMPP
NAD^+^-dependent glycerol-3-phosphate dehydrogenase (GPDH; EC: 1.1.1.8)	Glycerate 3-phosphate + NAD^+^ → Phosphoenolpyruvate + NADH	EMPP
NAD(P)^+^-dependent isocitrate dehydrogenase (IDH; EC:1.1.1.41, 1.1.1.42)	Isocitrate + NAD(P)^+^ → α-ketoglutarate + CO_2_ + NAD(P)H + H^+^	TCA cycle
NAD(P)^+^-specific malate dehydrogenase (ME; EC:1.1.1.37, 1.1.1.40)	Malate + NADP^+^ → pyruvate + CO_2_ + NADPH; malate + NAD^+^ → oxaloacetate + NADH + H^+^	TCA cycle, Anaplerotic node
α-Ketoglutarate dehydrogenase complex (αKGDH; EC: 1.2.4.2, 2.3.1.6, 1.8.1.4)	α-ketoglutarate + NAD^+^ + CoA → Succinyl CoA + CO_2_ + NADH	TCA cycle
Pyruvate dehydrogenase complex (PDC; EC: 1.2.4.1)	Pyruvate + NAD^+^ + CoA → Acetyl-CoA + NADH + CO_2_	EMPP, TCA cycle
NAD^+^-D-xylose dehydrogenase (XDH; EC: 1.1.1.175)	D-xylose + NAD^+^ → D-xylonolactone + NADH + H^+^	Dahms pathway

### 3.1 Engineering the Glycolytic and the Pentose Phosphate Pathways

#### 3.1.1 Overexpression of PPP Genes

Metabolic engineering to increase the NADPH yield per glucose consumed focused on the overexpression of the PPP genes *zwf* (glucose 6-phosphate dehydrogenase, G6PDH, EC 1.1.1.49) and *gnd* (6-phosphogluconate dehydrogenase, 6PGD, EC 1.1.1.44) both encoding NADPH forming enzymes (Lim et al., 2002; Poulsen et al., 2005) and/or the deletion of the glycolytic genes *pgi* (glucose-6-phosphate isomerase, GPI, EC 5.3.1.9) and *pfkA/B* (phosphofructokinase I/II, PFK, EC 2.7.1.11) (Canonaco et al., 2001; Lee et al., 2010) (Figure 2). Fuhrer et al. (2005) estimated that in growing *E. coli* cells, 12% of the glucose is catabolized *via* the PPP. The deletion of *pgi* prohibits glucose catabolism *via* glycolysis and enforces catabolism via the PPP, with 2 mol NADPH mol^−1^ glucose in a linear, non-cyclic fashion, plus the NADPH regenerated in the TCA cycle. Complete oxidation of glucose *via* a cyclic PPP requires complete cycling of both fructose 6-phosphate and triose phosphate and theoretically yields 12 mol NADPH per mole glucose (Kruger and von Schaewen, 2003). The ∆*pgi* mutant shows slower glucose uptake and a growth defect probably due to limited PPP flux capacity or regulatory effects on enzymes involved in glucose uptake or the PPP (Fuhrer et al., 2005). While deletion of *pgi* renders cyclization impossible, deletions in *pfkAB* redirect fructose 6-phosphate into the PPP further promoting catabolism *via* the PPP (Siedler et al., 2011).

Overexpression of *zwf* and *gnd* also was found to enhance NADPH-dependent PHB synthesis by recombinant *E. coli*, with *zwf* overexpression being three times more effective than *gnd* overexpression for increasing the NADPH (Lim et al., 2002). Lee et al. (2007) produced ɛ-caprolactone from cyclohexanone using whole cells of recombinant *E. coli* expressing cyclohexanone monooxygenase of *Acinetobacter calcoaceticus*. The maximum ɛ-caprolactone concentration was achieved by coexpression of the *zwf* gene enabling a 39% enhancement compared with the control. For (R)-3-hydroxybutyrate (3HB) production, Perez-Zabaleta et al. (2016) introduced the 3HB pathway of *Halomonas boliviensis* into *E. coli*, involving acetoacetyl-CoA reductase, which accepts both NADH and NADPH. The V_max_ for the latter is however eight times higher so that the less abundant NADPH limits production. Overexpression of *zwf* together with nitrogen depletion gave the highest yield and a 50% increase in 3HB concentration. Overexpression of PPP genes also has successfully been applied in organisms other than *E. coli*. For instance, the production of the poly-γ-glutamic acid (γ-PGA), a multifunctional non-toxic, biodegradable biopolymer, in *Bacillus licheniformis* was improved by 35% when *zwf* was overexpressed (Cai et al., 2017). In *Aspergillus niger*, 13-fold 6PGD overproduction increased the intracellular NADPH concentration 2- to 9-fold (Poulsen et al., 2005). It should however be noted that the overexpression of PPP genes may compromise target product formation if the substrate is used for growth, cofactor regeneration, as well as product formation as one carbon atom is lost *via* decarboxylation (6PGD-catalyzed reaction). In the case of NADPH-dependent GDP-L-fucose biosynthesis from glucose, for instance, *zwf* overexpression decreased the production under glucose-limiting condition even though the intracellular NADPH level was increased compared to the control strain. Fine-tuning of the glucose feeding strategy improved the GDP-L-fucose production by 21% with *zwf* overexpression, highlighting that sufficient supplementation of the carbon and energy source is critical when the PPP is engineered for NADPH-dependent biotransformations (Lee et al., 2011; Lee et al., 2013).

#### 3.1.2 Redirection of Glycolytic Fluxes to the PPP

Apart from overexpressing *zwf* and *gnd*, deletion of *pgi*, *pfkA*, and/or *pfkB* can also redirect the metabolic flux from glycolysis into the PPP (Figure 2) and has been employed in the NADPH-dependent production of various chemicals (e.g., hydrogen, amino acids, lycopene, 2-chloropropionic acid, terpenoids) (Aslan et al., 2017). Canonaco et al. (2001) showed via ^13^C-MFA that glucose catabolism in *E. coli* Δ*pgi* proceeds predominantly (almost 100%) *via* the PPP. Using a theoretical approach, the NADPH production rate was proposed to increase by 300% upon *pgi* deletion (Chemler et al., 2010). The complete flux through the PPP in the Δ*pgi* mutant was also independent of the growth phase in batch cultivation (Toya et al., 2010). In thymidine production, an important precursor of various antiviral drugs, NADPH is used not only for the conversion of uridine diphosphate to deoxyuridine monophosphate, but also for the recycling of tetrahydrofolate to dihydrofolate, a co-substrate for thymidine biosynthesis. Similarly, to *zwf* overexpression, Lee et al. (2010) reported that *pgi* deletion resulted in a 39% higher NADPH/NADP^+^ ratio which led to a 5-fold enhancement in thymidine yield on glucose. In the same manner, the NADPH-dependent production οf leucocyanidin and catechin from dihydroquercetin increased upon *pgi* deletion in *E. coli* (Chemler et al., 2010).

Deletions in *pfkAB* theoretically can afford even higher NADPH yields on glucose by recycling fructose 6-phosphate to glucose 6-phosphate (Kruger and von Schaewen, 2003). This partial cycling involves catabolism of PPP-derived glyceraldehyde 3-phosphate (G3P) *via* the lower part of glycolysis (EMPP) and the TCA cycle. Under resting cell conditions, this flux through EMPP and TCA cycle is necessary and beneficial for energy generation and PTS-driven glucose uptake (Siedler et al., 2011). For the production of methyl 3-hydroxybutyrate (MHB), a building block for widely used cholesterol-lowering statins (Poulsen et al., 2005), the prochiral β-ketoester methyl acetoacetate was reduced in recombinant *E. coli* by the NADPH-dependent R-specific alcohol dehydrogenase (ADH) from *L. brevis*. The MHB yield on glucose was almost doubled *via pfk*A deletion, which was attributed to partial PPP cyclization, which was confirmed by ^13^C-MFA revealing a negative net flux *via* PGI (Siedler et al., 2012). For further process optimization, Siedler et al. (2012) overexpressed the glucose facilitator and glucokinase genes of *Z. mobilis* and deleted either *pgi*, *pfk*A, or *pfk*A plus *pfk*B. In all cases, the glucose uptake rate increased (30–47%), and, for the Δ*pgi* and Δ*pfk*A strains, the specific MHB production rate increased by 15 and 20%, respectively. Siedler et al. (2013) also investigated *pfk*A or *gap*A (encoding G3P dehydrogenase) deletion in ADH carrying *C. glutamicum*. The WT showed a specific MHB production rate of 3.1 mmol g_CDW_^−1^ h^−1^ and a MHB yield of 2.7 mol per mol glucose, whereas the ∆*pfkA* mutant showed a similar rate, but a yield of 4.8 mol mol^−1^. The specific activity of the Δ*gapA* mutant decreased by 62% compared to the other strains, but the MHB yield increased to 7.9 mol per mol glucose.

Likewise, the PPP of *Streptomyces*, a genus widely used for the synthesis of various antibiotics, was successfully modulated. Borodina et al. (2008) deleted the *pfkA2* gene, one of the three annotated *pfkA* homologues in *S. coelicolor* A3, for the overproduction of the pigmented antibiotics actinorhodin and undecylprodigiosin. The *pfkA2* deletion increased the carbon flux through the PPP, as measured by ^13^C-MFA, involving increased intracellular glucose 6-phosphate and fructose 6-phosphate levels. Similarly, PPP flux was enhanced by deleting *gap*1 and *gap*2 *in S. clavuligerus*, thereby increasing the supply of the precursor G3P for the production of clavulanic acid, a β-lactamase inhibitor (Li and Townsend, 2006).

It is well reported that the NADPH supply, e.g., *via* the PPP, also is crucial for lipid and isoprenoid production by yeast (Zu et al., 2020; Lazar et al., 2018; Qiao et al., 2017; Wasylenko et al., 2015; Runguphan and Keasling, 2014). Apart from diverting carbon flux from glycolysis to the PPP through genetic manipulations (Figure 2), medium engineering (i.e., synergistic cofeeding) is also reported to be effective. Park et al. (2019) report that the coordination of carbons, ATP, and reduction equivalents required for lipid synthesis can be optimized by feeding substrate mixtures fueling diverse pathways. Controlled cofeeding of superior ATP and NADPH generating substrates, i.e., glucose to support H_2_-driven CO_2_ reduction and gluconate to drive acetate reduction, enabled not only circumvention of catabolite repression, but also synergistic co-metabolism in two divergent organisms, i.e., *Moorella thermoacetica* and *Yarrowia lipolytica*. Glucose doping of *M. thermoacetica* stimulated CO_2_ reduction into acetate by augmenting ATP synthesis *via* pyruvate kinase (PEP + ADP → pyruvate + ATP), and gluconate doping of *Y. lipolytica* accelerated acetate-driven lipogenesis by NADPH synthesis through cyclic PPP. Together, synergistic cofeeding was calculated to produce CO_2_-derived lipids with up to 38% energy yield (referred to H_2_ energy). Their stoichiometric analysis suggested that glucose and gluconate complemented ATP and NADPH generation, alleviating limitations seen in acetogenesis and lipogenesis, respectively. Tracing ^13^C-labelled glucose and gluconate revealed that cells used these substrates almost exclusively for ATP and NADPH production locally within glycolysis and the PPP, respectively. In particular, PPP activation by gluconate cofeeding solved the challenge of NADPH limitation in acetate-fed cells.

It should be noted that strategies involving a PPP knockout in combination with attenuated glycolysis can also be beneficial for biosynthetic processes, e.g., hyaluronic acid production. Based on a genome-scale metabolic model and respective simulation, Cheng et al., constructed a superior *C. glutamicum* strain for high-titer hyaluronic acid biosynthesis (Cheng et al., 2019). Genetic modulations included enhancement of the hyaluronic acid biosynthesis pathway, *zwf* deletion, fructose bisphosphate aldolase (FBA) attenuation, lactate/acetate pathway knockout, and pyruvate dehydrogenase complex (PDH) attenuation to drive carbon flux from the precursor metabolites glucose-6-phosphate (G6P), fructose-6-phosphate (F6P), and byproducts (lactate, acetate, succinate) to hyaluronic acid biosynthesis. The engineered *C. glutamicum* strain produced 28.7 g L^−1^ HA in a fed-batch culture, while the byproduct concentration was reduced by half.

#### 3.1.3 Switching the Cofactor Preference of Glycolytic Enzymes

Since the first report on redesigning the coenzyme specificity of a dehydrogenase by directed mutagenesis and molecular modelling (Scrutton et al., 1990), switching cofactor preference has been a target not only for enzymatic biocatalysis but also for engineering cellular metabolism towards increased product yields. The case of ethanol production from pentoses by *Saccharomyces cerevisiae* is one of the most studied examples of how switching the cofactor dependency of the involved metabolic pathways can enhance product yield and decrease unwanted side products such as xylitol and CO_2_ (Jeppsson et al., 2002; Verho et al., 2003; Petschacher and Nidetzky, 2008; Bengtsson et al., 2009). Apart from microbial platforms for ethanol or other fermentation product formation, switching cofactor preference of glycolytic enzymes also was applied for the bioproduction of industrially relevant terpenoids. These secondary metabolites are synthesized in dedicated biosynthetic pathways linked to the primary metabolism by specific precursors and various cofactors. Kim et al. (2018) engineered *S. cerevisiae* for the production of protopanaxadiol, a type of aglycone ginsenoside, by introducing the respective biosynthetic pathway and rerouting the redox metabolism to improve NADPH availability. By replacing the cytoplasmic NAD^+^-utilizing aldehyde dehydrogenase converting acetaldehyde to acetate with a functionally equivalent NADP^+^-dependent isozyme (Figure 2), the final protopanaxadiol titer could be increased more than 11-fold (Kim et al., 2018). Kildegaard et al. (2016) engineered *S. cerevisiae* for high-level 3-hydroxypropionic acid (3HP) production by increasing biosynthetic gene copy numbers and improving flux towards precursors and redox cofactors. 3HP was produced *via* a malonyl-CoA reductase (MCR) that reduces malonyl-CoA to 3HP at the expense of two NADPH. Genomic integration of multiple copies of *Chloroflexus aurantiacus* MCR and phosphorylation-deficient acetyl-CoA carboxylase genes increased the 3HP titer 5-fold in comparison with single integration. Furthermore, the native NAD^+^-dependent glyceraldehyde-3-phosphate dehydrogenase (GAPDH) was exchanged with a heterologous NADP^+^-dependent version from *Chlostridium acetobutylicum* to increase NADPH formation (Figure 2) and thus improve 3HP production. However, even though the two major pathways for NADPH regeneration (i.e., PPP and TCA cycle) were upregulated and carried higher flux in the engineered strain, both NADPH/NADP^+^ as well as NADH/NAD^+^ ratios increased, underlining the complexity of redox metabolism (Kildegaard et al., 2016). The same GAPDH exchange was reported to enhance lycopene and ε-caprolactone production in *E. coli* (Martínez et al., 2008).

#### 3.1.4 Engineering the Entner–Doudoroff Pathway

Microorganisms utilize three major pathways to break down glucose into pyruvate, i.e., EMPP, EDP, and oxidative PPP (OPPP) (Figure 2). *E. coli* mainly relies on the EMPP and the OPPP, while the EDP is mainly inactive except during growth on gluconate. Interestingly, the EDP was found to be active in the cyanobacterium *Synechocystis* sp. PCC 6803, whose nutrient- rather than ATP-limited lifestyle profits from the lower costs for enzyme synthesis (Chen et al., 2016). In these phototrophs, the EDP seems to be important for the internal glycogen breakdown under fluctuating carbon availability (Lucius et al., 2021). Physiological analyses of different glycolytic strategies revealed that the EDP could be a preferred pathway based on both thermodynamics and the considerably lower enzyme costs. The EMPP operates 10 enzymatic reactions to yield two pyruvates, two ATP, and two NADH per glucose molecule, the OPPP provides two NADPH at the expense of one carbon atom lost as CO_2_, and the EDP operates five enzymes to produce one pyruvate, one glyceraldehyde-3-phosphate, one ATP, one NADH and one NADPH per glucose molecule often constituting a well-balanced product mix. Further, the EDP bypasses the two thermodynamic bottlenecks of the EMPP, i.e., fructose 1,6-bisphosphate aldolase and triose-phosphate isomerase but at the expense of ATP yield. This characterizes EMPP and EDP as routes with high protein synthesis costs and poor ATP yield, respectively (Hollinshead et al., 2016). With an active EMPP, GAPDH is the enzyme showing the highest NADH formation rate during aerobic as well as anaerobic growth of *E. coli*. Many organisms lack this route and use the EDP instead with both GAPDH and glucose-6-phosphate dehydrogenase showing high rates (Schrewe et al., 2013). EDP-derived NADPH supports oxidative stress responses and, in contrast to PPP-derived NADPH, is formed without carbon loss (i.e., CO_2_ formation) (Hollinshead et al., 2016; Jojima et al., 2021). Recently, EDP upregulation in *E. coli* was shown to enhance glucose consumption as well as G3P and pyruvate formation serving as precursors of isoprenoid synthesis *via* the methylerythritol 4-phosphate (MEP) pathway (Jojima et al., 2021). The latter depends on high and balanced flux among precursors, cofactors, and cellular energy and is limited by the imbalanced supply of G3P and pyruvate in *E. coli*. To address this problem, Li et al. (2015b) either knocked out or overexpressed multiple gene targets to redistribute fluxes among EMPP, EDP, and PPP and thus improve carotenoid production. Directing metabolic flux from EMPP towards EDP together with an enhanced MEP pathway improved carotenoid synthesis, which was further improved by *pgi* deletion. On the contrary, *pfkAB* deletion had a negative effect. Whereas *pgi* deletion blocks the recycling of F6P and G3P into the OPPP and supports flux towards the MEP substrates pyruvate and G3P, *pfkAB* deletion enables F6P cyclization into the PPP. Importantly, improved carotenoid yields were accompanied by increased biomass growth and decreased acetate overflow, indicating a good balance between carotenoid biosynthesis and cell metabolism (Li et al., 2015b).

In a similar attempt to improve isoprenoid biosynthesis from glucose and xylose in *E. coli*, Liu et al. (2013) evaluated EMPP, EDP, PPP, as well as the Dahms pathway as MEP feeding modules differing in their modes of G3P, pyruvate, and reducing equivalent generation. In EMPP and PPP, G3P generation precedes pyruvate formation, whereas the Dahms pathway generates pyruvate from G3P. Only the EDP simultaneously produces the two MEP precursors in one reaction. Although lower amounts of precursors are produced when glucose is metabolized via the PPP, more reducing equivalents can be generated compared to EMPP and EDP. From glucose, feeding modules containing the EDP enabled three and six times higher isoprene titers and yields, respectively, compared to the EMPP. From xylose, the PPP module was significantly more effective than the Dahms pathway. In terms of precursor generation and energy/reducing-equivalent supply from glucose, the EDP together with the PPP was found to be the optimal module combination (Liu et al., 2013).

Furthermore, OPPP and EDP were investigated as engineering targets for mevalonate production. In an *E. coli* strain expressing the *Enterococcus faecalis* genes *mvaE* and *mvaS*, one mevalonate molecule is synthesized from three acetyl-CoA molecules using two molecules of NADPH in a 3-step reaction. Nagai et al. studied thermodynamic states of the central metabolism to identify metabolic reactions with regulatory function. Based on metabolite concentration data, they calculated ΔG and substrate saturation for each metabolic reaction, comparing control and mevalonate producing strains. Their analyses showed that further activation of thermodynamically feasible reactions in the upper part of glycolysis and the PPP appeared difficult and that metabolic bypassing *via* the EDP was a promising strategy to increase acetyl-CoA and NADPH supply. Deletion of *gnd* and *gntR* (encoding a negative regulator of the expression of two EDP genes) increased mevalonate yield and specific production rate by 113 and 158%, respectively (Nagai et al., 2018). Redirection to the EDP has also been employed for the production of isopropyl alcohol (Okahashi et al., 2017), poly(3-hydroxybutyrate) (Zhang et al., 2014), and isobutanol (Noda et al., 2019) with *E. coli*. Instead of using existing operons, Ng et al. (2015), rationally engineered synthetic operons for the 5-enzyme pathway of *Z. mobilis* and increased the NADPH regeneration rate 25-fold. By combining the synthetic EDP with an optimized terpenoid pathway, the terpenoid titer was increased by 97% (Ng et al., 2015). These successful attempts clearly show that even though microbial cells prefer EMPP for optimal growth, metabolic engineering strategies involving the EDP appear very promising to improve product formation rates and yields.

### 3.2 Increasing the Availability of Redox Cofactors and Cosubstrates Derived From the TCA Cycle

The TCA cycle is the primary metabolic pathway enabling aerobic organisms to oxidize diverse organic compounds, such as carbohydrates, lipids, or amino acids, to provide redox equivalents, energy, and precursors for biomass formation to the cell. Biotransformations can profit from the TCA cycle for the provision not only of redox cofactors but also of TCA intermediates as cosubstrates, as in the case of α-ketoglutarate (α-KG) dependent dioxygenases (Figure 2). Here, it has to be considered that the TCA cycle of cyanobacteria is different from that of heterotrophic bacteria (Knoop et al., 2013; Xiong et al., 2014; Zhang et al., 2016b), as it lacks α-KG dehydrogenase and thus cannot convert α-KG to succinyl–CoA. The cycle was however shown to be completed by two alternative enzymes, a novel α-KG decarboxylase and a succinic semialdehyde dehydrogenase. This corrected the misconception that these organisms have an incomplete TCA cycle and shed light on new metabolic potentials for biotechnological exploitation (Zhang et al., 2016b). The synthesis of *trans*-4-hydroxy-L-proline by both heterotrophic and photosynthetic cells using an α-KG dependent L-proline-4-hydroxylase is such a successful example (Falcioni et al., 2015; Theodosiou et al., 2017; Zhang et al., 2021a; Brandenburg et al., 2021). This and other examples for TCA dependent biocatalytic reactions are discussed in the following.

#### 3.2.1 Engineering Redox Cofactor Supply *via* TCA Cycle and Anaplerotic Pathways

Apart from the PPP and the proton-translocating transhydrogenase PntAB, the malic enzyme (ME) is also considered a major source of NAD(P)H and a promising target for redox metabolism engineering (Figure 2). ME catalyzes the reversible oxidative decarboxylation of malate to pyruvate. MEs can reduce NAD^+^, NADP^+^, or both cofactors. In *E. coli*, there are 2 genes, i.e., *maeA* (encoding a NAD^+^-ME) and *maeB* (encoding a NADP^+^-ME), which are part of the anaplerotic node that links glycolysis and gluconeogenesis to the TCA cycle (Sauer and Eikmanns, 2005). Isocitrate dehydrogenase (*icd*) can also be an important NAD(P)H supplier (Sauer et al., 2004). Whereas *E. coli* IDH depends on NADP^+^, eukaryotes also contain a NAD^+^-dependent IDH playing an important role in the TCA cycle (Cupp and McAlister-Henn, 1991). Finally, the glyoxylate shunt can have an effect on the NADPH generation and is often manipulated to redirect fluxes. It bypasses the conversion of isocitrate to succinate and respective NAD(P)H generation in the TCA cycle. When the glyoxylate shunt is activated (e.g., by deletion of the respective repressor gene *iclR*), more malate is formed, which in turn can be converted to pyruvate by the ME involving NADPH formation (Lin et al., 2013).

Lee et al. (2011) overexpressed *zwf*, *icd*, and *maeB* genes in order to improve NADPH-dependent GDP-l-fucose productivity in recombinant *E. coli* bearing GDP-l-fucose biosynthetic enzymes. Overexpression of *icd* resulted in 30% higher GDP-L-fucose production compared with the control, similarly as *zwf* overexpression. Overexpression of *maeB* however resulted in a 24% reduction of GDP-L-fucose production. Liu et al. (2018) overexpressed isocitrate dehydrogenase and citrate synthase genes in *B. licheniformis* DW2 to allow a high TCA cycle flux and study whether the improved ATP and NADPH levels could concomitantly enhance bacitracin biosynthesis. What they found was that the increased TCA cycle flux resulted in more energy generation and improved amino acid uptake and intracellular biosynthesis leading to larger pools of precursor amino acids for bacitracin biosynthesis. The contribution of the TCA cycle in NADPH regeneration was also confirmed by Chin et al. (2009) who worked with *E. coli* bearing NADPH-dependent xylose reductase from *C. boidinii*. They reported that succinyl-CoA synthetase (*sucC*) deletion significantly reduced the xylitol yield on glucose provided as cosubstrate, indicating that a functional TCA cycle contributes to NADPH allocation for xylitol production.

ME has a decisive role as major NADPH provider for fatty acid biosynthesis in oleaginous microorganisms and is often considered rate-limiting (Liang and Jiang, 2013; Ratledge, 2014). It was shown that *maeB* overexpression in *Mucor cicinelloides*, a commercial oil-producing fungus, resulted in a 2.5-fold increase in lipid accumulation (Zhang et al., 2007). This *mae*B gene also was overexpressed in the oleaginous yeast *Rhodotorula glutinis* and improved its lipid content by more than 2-fold (from 18.74 to 39.35% w/w) without affecting fatty acid profiles (Li et al., 2013). *E. coli* could be an alternative fatty acid producer even though it provides only a small amount of the precursor malonyl-CoA. Meng et al. (2011) simulated the lipogenesis of oleaginous microorganisms in *E. coli* by coexpressing the acetyl-CoA carboxylase genes (*accABCD*) from *A. calcoaceticus* to improve malonyl-CoA formation and the *maeB* gene from *E. coli* to improve NADPH availability. The overexpression of the *acc* genes alone improved malonyl-CoA formation resulting in a 3-fold increase in intracellular lipids, whereas *maeB* overexpression together with the addition of malate resulted in a 4-fold increase in intracellular lipids (197.74 mg g^−1^). Combining the approaches, a 5.6-fold increase of intracellular lipids was achieved (284.56 mg g^−1^). Similarly, the overexpression of ME from the oleaginous fungus *Mortierell alpine* in fatty alcohol producing *S. cerevisiae* strains lead to increased final fatty alcohol titers, with the reduction of fatty acyl-CoA to fatty alcohol requiring stoichiometric amounts of NADPH (Runguphan and Keasling, 2014).

These studies implicate ME to be a key supplier of lipogenic NADPH. However, ^13^C-MFA by Wasylenko et al. revealed that, in engineered *Y. lipolytica* showing a two-fold increased fatty acid yield compared to the control strain, NADPH formation via the oxidative PPP was approximately doubled while the ME flux did not differ significantly between the two strains (Wasylenko et al., 2015). Moreover, they found that the PPP-related NADPH formation rate was in good agreement with the estimated NADPH demand for fatty acid biosynthesis in both strains, identifying the oxidative PPP as the primary source of lipogenic NADPH in *Y. lipolytica*. Based on this finding, Qiao et al. increased lipid synthesis by engineering pathways in *Y. lipolytica* that utilize glycolytic NADH to promote the synthesis of the lipid precursors NADPH or acetyl-CoA (Qiao et al., 2017)*.* For this purpose, they constructed 13 novel strains based on a *Y. lipolytica* variant with overexpressed acetyl-CoA carboxylase and diacylglyceride acyltransferase. They concluded that at least three strategies can be applied to convert most of the cytosolic NADH to NADPH leading to overall process yield improvement: 1) high-level expression of the NADP^+^-dependent glyceraldhyde-3-phosphate dehydrogenase (GAPDH) from *C. acetobutylicum*, 2) overexpression of NAD^+^ kinase, and 3) introduction of cytosolic NADP^+^-dependent malic enzyme. Interestingly, the conversion of NADH to NADPH reduced the O_2_ demand of the culture enabling higher cell densities with the same aeration capacity.

MFA has shown that the competition for NAD(P)H between redox biocatalysis and the energy metabolism becomes critical during asymmetric styrene epoxidation catalyzed by growing *E. coli* containing recombinant NADH-dependent styrene monooxygenase (Bühler et al., 2008). Engineering TCA-cycle regulation allowed increased TCA-cycle activities, a delay of acetate formation, and enhanced NAD(P)H yields during batch cultivation (Kuhn et al., 2013). This, however, could not improve whole-cell styrene epoxidation activities, whereas elevated product concentrations were found to cause a significantly increased NAD(P)H demand and a compromised efficiency of metabolic operation. Higher styrene epoxidation activities however were obtained with constitutively solvent-tolerant *Pseudomonas taiwanensis* VLB120ΔCΔ*ttgV* (Volmer et al., 2014) as host strain with an inherently higher glucose uptake rate and TCA cycle activity (Volmer et al., 2019).

#### 3.2.2 Engineering Cosubstrate Supply *via* the TCA Cycle

As mentioned above, synthetically interesting biocatalytic reactions can directly depend on central carbon metabolites that serve as cosubstrates. The hydroxylation of L-proline to trans-4-hydroxy-L-proline (hyp) using an α-KG dependent L-proline-4-hydroxylase (P4H) is such an example (Shibasaki et al., 2000). The reaction catalyzed by recombinant *E. coli* cells bearing P4H from *Dactylosporangium* sp. strain RH1 requires stoichiometric amounts of the TCA cycle intermediate α-KG. In this hydroxylation reaction, one atom of molecular oxygen is introduced into L-proline to yield hyp, while the other oxygen atom is used for oxidative decarboxylation of α-KG giving rise to succinate and CO_2_. This decarboxylation added a shortcut of the TCA. For the synthesis of hyp from proline, a commercially used whole-cell process has been reported (Shibasaki et al., 2000) and the interdependency of process conditions, host metabolism, and catalyst performance was resolved (Theodosiou et al., 2015). In an attempt to evaluate TCA cycle engineering strategies to enforce and increase α-KG flux through P4H, Theodosiou et al. (2017) deleted the TCA-cycle genes *sucA* (*α*-KG dehydrogenase E1 subunit) or *sucC* (succinyl-CoA synthetase β subunit) together with *aceA* (isocitrate lyase), disrupting both glyoxylate shunt and TCA cycle (Figure 2), in a proline degradation-deficient *E. coli* strain (Δ*putA*) expressing the *p4h* gene. Whereas the Δ*sucC*Δ*aceA*Δ*putA* strain grew in minimal medium in the absence of P4H, relying on the activity of fumarate reductase, growth of the Δ*sucA*Δ*aceA*Δ*putA* strictly depended on P4H activity, thus coupling growth to proline hydroxylation. Employing a Na^+^/L-proline transporter (*putP* gene) in the Δ*sucA*Δ*aceA*Δ*putA* strain*,* the specific proline hydroxylation rate doubled (Theodosiou et al., 2017). In a similar approach, Smirnov et al. (2010) produced (2S,3R, 4S)-4-hydroxyisoleucine from L-isoleucine by means of recombinant *E. coli* carrying an α-ketoglutarate-dependent L-isoleucine dioxygenase (IDO) (Kodera et al., 2009). Also here, IDO introduction was able to shunt the TCA cycle in an *E. coli* mutant with both glyoxylate shunt and TCA-cycle disrupted, forcing metabolic flux through the hydroxylation reaction. The same engineering was successfully applied for the synthesis of G-7-aminodeacetoxycephalosporanic acid (G-7-ADCA) from penicillin G (Lin et al., 2015). In that case, an *E. coli* strain expressing α-KG-dependent deacetoxycephalosporin C synthase (DAOCS) was used to reconstitute the TCA cycle. Additional engineering (*poxB* deletion and *acs* overexpression) reduced acetate accumulation in order to prevent medium acidification and carbon loss. Together with the deletion of the host *β*-lactamase involved in penicillin G and G-7-ADCA degradation, the G-7-ADCA titer could be increased 11-fold to 29.01 mM. Interestingly, the constructed vector systems did not require antibiotic selection, as survival depended on the expression of the dioxygenase encoded on the vector.

**FIGURE 3:**
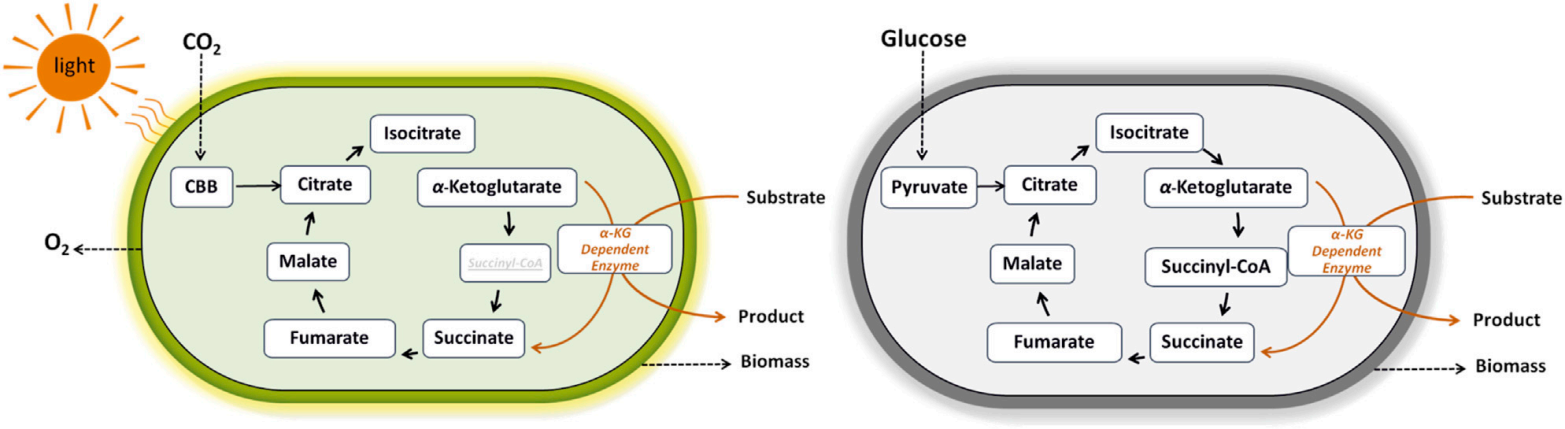
Coupling of α-ketoglutarate dependent oxygenases to phototrophic and heterotrophic metabolism. These enzymes catalyze a remarkable range of reactions, such as hydroxylation, desaturation, and epoxidation. They utilize iron (II) as the metallo-cofactor and α-ketoglutarate as the co-substrate that is oxidatively decarboxylated to succinate and CO_2_.

Whilethe exploitation of α-KG dependent dioxygenases in heterotrophic bacteria requires organic carbon to fuel the TCA cycle, cyanobacterial reliance on CO_2_ offers a promising sustainable alternative (Figure 3). The first exploitation of the cyanobacterial TCA cycle was reported in 1994 for the production of ethylene, a widely used feedstock in plastic industry, by introducing the *efe* gene from *Pseudomonas syringae pv. phaseolicola* PK2 into *S. elongatus* PCC 7942 (Fukuda et al., 1994). Since then, metabolic engineering and synthetic biology of cyanobacteria greatly advanced photoautotrophic ethylene production as recently reviewed by Kallio et al. (2021). Regarding amino acid hydroxylation with high potential in food and pharmaceutical industries, the work of Brandenburg et al. (2021) describes the first successful example of photosynthetic hyp production directly from CO_2_, by genetic engineering of the cyanobacterium *Synechocystis* sp. PCC 6803 bearing the *p4h* gene from *Dactylosporangium*.

### 3.3 Engineering Phototrophic Metabolic Modules for Whole-Cell Biocatalysis

#### 3.3.1 Direct Utilization of Light

**FIGURE 4:**
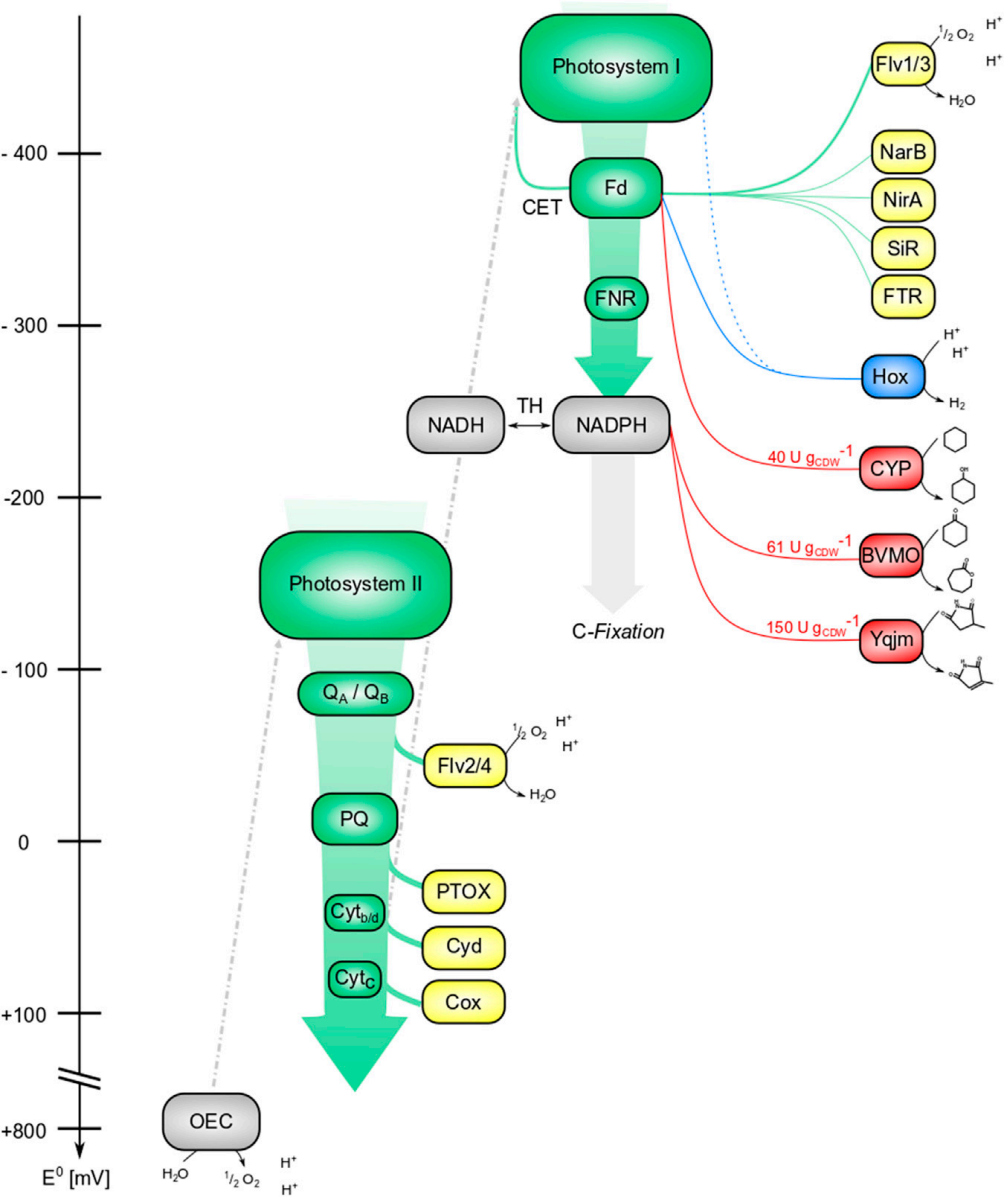
Tapping the reductive potential of photosynthesis. Green: components of the photosynthetic electron transfer chain (PETC); yellow: internal electron sinks; blue: (native) hydrogenase (Hox); red: (recombinant) redox enzymes. Electrons derived from water splitting enter the photosynthetic electron transport chain at photosystem II, where energy from light quanta promotes them to a higher excitation state (a more negative potential). This excited state can be used for photochemistry, i.e., the reduction of the primary acceptor Q_A_/Q_B_, followed by electron transport over plastoquinone (PQ) and cytochromes (Cyt_b/d_ and Cyt_c_) along a gradient of redox potentials. Photosystem I again absorbs light energy and constitutes a reducing agent strong enough to transfer electrons to Ferredoxin (Fd). The minimal redox potentials in photosystems are not drawn to scale. Ferredoxin-NADPH-reductase (FNR) allocates a big share of these electrons to NADPH, which either can be used for C-fixation or can be converted to NADH by transhydrogenases (TH). Alternatively, electrons can be distributed to N- and S-assimilation (Fd-nitrate-reductase NarB, Fd-nitrite-reductase NirA, and Fd-sulfite-reductase SiR), regulatory elements (e.g., Fd-thioredoxin-reductase FTR), or back to photosystem I, referred to as cyclic electron transfer (CET). At different points of the PETC, reductive stress can be released by transferring electrons to acceptors like the flavodiiron proteins Flv2/4 and Flv1/3 and respiratory oxidases like the PQ-terminal oxidase PTOX, Cyt_b/d_- oxidase Cyd, and Cyt_c_-oxidase Cox. Redox potentials of electron sinks are not drawn to scale. So far, the reductive potential of photosynthesis was tapped at or downstream of PS I, by fusion constructs (dotted line), at the level of Fd (Hox and CYP450), and at the level of NADPH (Baeyer-Villiger monooxygenase BVMO and ene-reductase Yqjm).

Biocatalytic redox reactions depend not only on suitable enzymes, but also on efficient and sustainable redox cofactor supply. To find the optimal electron transfer partner, both the redox potential and the supply rate of this carrier have to be considered. Most commonly, NADH- or NADPH-dependent enzymes are used. However, photosynthetic cells offer various other electron carriers, such as ferredoxin (Fd), the primary electron acceptor of photosystem I. Figure 4 displays the photosynthetic electron transport chain (PETC) and the variety of electron carriers involved as well as possible homo-/heterologous electron sinks.

Due to the relatively low redox potential of Fd (Wlodarczyk et al., 2015; Berepiki et al., 2016; Hoschek et al., 2019a), application of Fd-dependent enzymes seems to be highly promising. Several Fd-dependent oxygenases have been applied and specific activities up to 40 U g_CDW_^−1^ were achieved. Both enzyme specific and host intrinsic Fds have been found to shuttle electrons, with differing efficiency. Phototrophs contain multiple Fds with, e.g., at least nine Fd in the model cyanobacterium *Synechocystis* sp. PCC 6803 (Cassier-Chauvat and Chauvat, 2014). This complicates the prediction of electron transfer efficiencies towards Fd-dependent enzymes. NADPH (Böhmer et al., 2017; Tüllinghoff et al., 2022), NADH, flavodoxins or plastochinones also have the potential to be used as electron donors. Enzymes depending on NADPH as a main product of the light reaction may benefit from the relatively large NADPH pool, but may suffer from competition with electron consuming metabolic routes, especially CO_2_ fixation and nitrate assimilation (Mellor et al., 2017). A study investigating the most suitable carrier for a CYP450 showed that 1) the type of available electron carrier determines the biocatalytic reaction rate and 2) this rate profits from electron carrier-CYP450 fusion (Mellor et al., 2019). Methods to improve intracellular CYP450 catalysis, including enzyme-, redox-partner-, and substrate engineering recently were nicely reviewed by Li et al. (2020). An improved connection to the PETC has the potential to significantly increase production rates, as shown for the linkage of a CYP450 to the thylakoid membrane (Lassen et al., 2014a). A direct connection to the photosynthetic apparatus, as demonstrated for hydrogenases (Appel et al., 2020; Kanygin et al., 2020), allowed a direct electron transfer, mitigating unwanted electron leakage. As a strategy to avoid intracellular competition for redox equivalents, FNR affinity for ferredoxin can be attenuated, thereby making more ferredoxin available for target reactions, e.g., hydrogen formation catalyzed by Fd-accepting hydrogenases (Kannchen et al., 2020). The plastochinone pool in principle constitutes another attractive point to tap reducing power from photosynthesis, with native transfer to the terminal oxidase as an example (Feilke et al., 2017). Its relatively positive redox potential however limits the amount of reactions to be potentially fueled by plastochinone.

The implementation of biocatalytic reactions in phototrophs mostly constituted an iterative process. Several CYP450, BVMOs, reductases, hydrogenases, etc., have been applied with specific activities up to 150 U g_CDW_^−1^ as summarized in Table 2. Interestingly, electron drainage *via* biotransformation reactions appeared to cause an ATP/NADPH imbalance, (possibly) affecting both the biocatalytic reaction and the host metabolism. The introduction of heterologous pathways relying on C-fixation and reducing power showed a way to avoid such imbalance (Berepiki et al., 2018; Santos-Merino et al., 2021). Thus, the balance of sources and sinks should be considered. As with heterotrophic microbes, substrate and product inhibition, toxicity, and volatility also have to be considered with substrate supply as a biochemical engineering target (Lin and Tao, 2017). For BVMO catalysis in *Synechocystis* sp. PCC 6803 for instance, kinetic constraints have been found to be more relevant and thus overlay photosynthesis-related effects such as light limitation (Tüllinghoff et al., 2022).

**TABLE 2:** Recombinant redox biotransformations in cyanobacteria and microalgae. Adapted from Jodlbauer et al. (2021) und updated.

Host	Enzyme	Tapping Point	Product	Max. Activity/*Scale*	References
*Synechocystis* sp. PCC 6803	Yqjm ene reductase (*Bacillus subtilis*)	NADPH	2-methyl-succinimide	150 U g_CDW_^−1^, *1 ml, 5–30 min*, 25.7 U g_CDW_^−1^ ^a^, *200 ml, 22 h*	Assil-Companioni et al. (2020), (Hobisch et al., 2021)
	Cytochrome P450 monoxygenase (*Acidovorax* sp. CHX100)	Fd	Cyclohexanol	39.2 U g_CDW_^−1^, *2 L, 52 h*	Hoschek et al. (2019a)
	Alkane monooxygenase (*Pseudomonas putida*)	NADH	H-NAME	1.5 U g_CDW_^−1^, *1 ml, 30 min*	Hoschek et al. (2017)
	Baeyer-Villiger-Monoxygenase (*Acidovorax* sp. CHX100)	NADPH	ε—Caprolactone	60.9 U g_CDW_^−1^, *2 L, 24 h*	Tüllinghoff et al. (2022)
	Imine reductase (*Streptomyces* sp. GF3587)	NADPH	Cyclic amines	21.8 U g_CDW_^−1^ ^a^	Büchsenschütz et al. (2020)
	Cytochrome P450 monoxygenase CYP79A1 and CYP71E1 and glycosyltransferase (*Sorghum bicolor*)	Fd	*p*-Hydroxyphenylacetaloxim	1.0 U g_CDW_^−1^	Wlodarczyk et al. (2015)
*Synechococcus elongatus* PCC 7942	Alcohol dehydrogenase (*Lactobacillus kefir*)	NADPH	Dhurrin 1-phenylethanol	0.03 U g_CDW_^−1^, 84 U g_CDW_^−1^ ^a^	Sengupta et al. (2019)
*Synechococcus* sp. PCC 7002	Cytochrome P450 monoxygenase CYP79A1 (*Sorghum bicolor*)	Fd	*p*-Hydroxyphenylacetaloxim	(26 µM titer with OD_730_ = 2.5)^d^	Lassen et al. (2014a)
*Chlamydomonas reinhardtii*	Amine dehydrogenase (*Exiguobacterium sibiricum*)	NADH^b^	Linear and cylcic amines	<0.5 U 10^−8^_cells_ ≈ 0.5 U g_CDW_^−1^ ^c^, *2 ml, 40 h*	Löwe et al. (2018)

a —Calculated from substrate consumption.

b —Regenerated by formate reduction by *C. reinhardtii.*

c —Estimation based on (Fan et al., 2017)*.*

d —Time of biotransformation not available.

H-NAME—ω-hydroxynanoic acid methyl ester, Fd—Ferredoxin.

In contrast to cyanobacteria, which have been used in various studies for biocatalysis, examples employing green algae are scarce (Zheng et al., 2018), which is mostly due to the limited molecular biology toolbox for such strains. *Chlorella vulgaris* or *Chlamydomonas reinhardtii* were recently applied as whole-cell biocatalysts, using the photosynthetic reduction power for either dehalogenations (Khan et al., 2021) or aliphatic amine formation via an amine dehydrogenase (Löwe et al., 2018; Gröger, 2019). The presented examples indicate a limited applicability of phototrophs for NADH-dependent reactions, which only indirectly couple to the photosynthetic light reaction. Application of such enzymes in cyanobacteria/green algae requires either a change in enzymatic cofactor preference (Park and Choi, 2017; Wang et al., 2017) or redirection of electrons towards NADH. Implementation of NADH-dependent nitrate assimilation led to enhanced NADH production, supporting the target pathways and finally the NADPH-dependent butanol formation (Purdy et al., 2022).

Besides providing reduction power, the photosynthetic light reaction can supply O_2_ and thus overcome one major limitation in the application of oxygenases in heterotrophs. Theoretical calculations indicate that optimally aerated growing heterotrophic cells can reach productivities up to 3.5 g L^−1^ h^−1^ without oxygen limitation (Pedersen et al., 2015). For CYP catalysis in cyanobacteria (Hoschek et al., 2018), external O_2_ supply could even be completely omitted and the reaction be fueled by photosynthesis-derived O_2_. Additionally, an indirect O_2_ supply was demonstrated in a mixed species approach, in which phototrophs provided heterotrophic whole-cell biocatalysts with O_2_ (Hoschek et al., 2019b; Heuschkel et al., 2019). Furthermore, photosynthetic *in situ* O_2_ supply makes application of other O_2_-dependent enzymes not relying on reduction power conceivable, e.g., alcohol oxidases, amine oxidases, laccases, and dioxygenases. The oxidation of alcohols to aldehydes or ketones by alcohol oxidases is of particular interest for the production of flavors and fragrances. Examples include benzaldehyde (bitter almond), cinnamaldehyde (cinnamon), octanal (citrus), 2-heptanone (banana), ionones (rose), and vanillin (vanilla) (Berger, 2009; Berger, 2015). Laccases, which gained interest in recent years (Bassanini et al., 2021), are present in phototrophic species and have the potential to be used for degradation processes (Afreen et al., 2017; Liang et al., 2018).

#### 3.3.2 Calculations Regarding Light-to-Product Efficiency

For the envisioned photosynthesis-driven redox biocatalysis, the light-to-product efficiency is of special interest. Major questions to be answered, are:

1) How efficiently does the biocatalyst use the incident light? What are the resulting electron transfer rates (ETRs)?
2) At which point can photosynthesis be tapped with what efficiency?
3) Which strains or strain character traits are desirable to improve photosynthesis-driven biotechnology?

The light-to-biomass efficiency is maximal at the light saturation point E_k_, where the incident light is just sufficient to supply the metabolism with the reductive power needed, e.g., for biomass production. In natural environments, phototrophic organisms face a “light usage dilemma” (Wilhelm et al., 2021), consisting of the incident light being either below or above E_k_, impairing overall photosynthetic efficiency. In fact, 82% of the incident photosynthetically active radiation (PAR) reach the surface with light intensities above E_k_ (Wilhelm et al., 2021). Consequently, the photosynthetic apparatus is evolutionarily optimized to handle over-excitation rather than to maximize light usage efficiency.

Within the photosynthetic apparatus, (excess) energy is lost at different levels, often by mechanisms important to protect the apparatus from over-excitation: 1) Depending on their pigmentation, photosynthetic organisms absorb only a share of PAR, expressed as the share of photosynthetically absorbed quanta Q_phar_. 2) At photosystem II (PSII), a part of the energy is dissipated via heat or fluorescence emission and non-photochemical quenching (NPQ), which decreases the operative quantum yield at PSII (Ф_PSII_). Ф_PSII_ can be assessed *via* pulse-amplitude-modulation (PAM) fluorometry to calculate the ETR in PSII. 3) Alternative electron flux (AEF) via terminal oxidases and Mehler- or Mehler-type reactions further compromises the light-to-biomass efficiency. As a result, only a fraction of the incident light energy can be used for biomass formation or productive (biocatalytic) reactions. To illustrate these theoretical considerations, Table 3 summarizes relevant rates of O_2_ evolution, electron supply, and growth for typical phototrophic microorganisms.

**TABLE 3:** Photosynthetic rates of phototrophic host organisms applied for redox biocatalysis.

Host	O_2_-evolution rate (mol O_2_ g_Chl a_^−1^ h^−1^)	e^−^ supply rate (mol e^−^ g_Chl a_^−1^ h^−1^)	µ_max_ (h^−1^)
*Synechocystis* sp. PCC 6803	0.34−1.01^a^ (Zavřel et al., 2019)	1.08–2.16^b^ (Kauny and Sétif, 2014)	0.135 (Zavrel et al., 2015)
*Synechococcus elongatus* UTEX 2973	0.82 (Ungerer et al., 2018)	—	0.365 (Yu et al., 2015)
*Chlamydomonas reinhardtii*	0.15 ± 0.01 (Langner et al., 2009)	1.08 ± 0.06^c^ (Langner et al., 2009)	0.059 (Boyle and Morgan, 2009)
*Chlorella vulgaris*	0.11 ± 0.01 (Wagner et al., 2006)	0.94 ± 0.02^c^ (Wagner et al., 2006)	0.07 (Schuurmans et al., 2015)

a Depending on light conditions.

b Assessed *via* NADPH-fluorescence measurements.

c Assessed *via* Chl_a_ measurements at PS II.

Due to the energy losses along the photosynthetic electron transfer chain, it is obvious that the closer a reaction is situated to the water splitting, the higher are electron supply rate and light use efficiencies. About 50–60% (Wilhelm et al., 2021) of the light energy can in principle be tapped at PSII or directly downstream to it, at the plastoquinone pool, where losses only result from heat and fluorescence dissipation and non-photochemical quenching. Tapping reductive power at PS I or at its acceptor side (*via* Fd or NADPH as described above) involves electron losses to respiratory elements, like oxidases, or Mehler- and Mehler-type reactions. Taken together, these losses account for 20–30%, reducing the light use efficiency to 15–20%. Beside these theoretical efficiencies, the redox potentials of tapped PETC cofactors or electron carriers are of practical importance as reviewed by Mellor et al. (2017): PSII [∼−110 mV in *Synechocystis* sp. PCC 6803 (Allakhverdiev et al., 2011)] and plastoquinone [+80 to +110 mV (Antal et al., 2013)] have a by far lower reductive power than already used electron carriers like Fd [−430 mV to −300 mV (Cammack et al., 1977)] and NADPH (−320 mV), which makes the latter ones highly suitable electron donors for redox biocatalysis.

In agreement with these theoretical considerations regarding light-to-product efficiencies, potential hosts for light-driven biotechnology require special character traits to ensure powerful and robust processes. High-light- and high-temperature-tolerances are especially important for outdoor applications. Adaptive laboratory evolution (ALE) in combination with systems biology approaches, were successfully applied for *Chlamydomonas, Synechocystis*, and *Synechococcus* (Zhang et al., 2021b; Srivastava et al., 2021; LaPanse et al., 2021; Dann et al., 2021; Mukherjee et al., 2020). *Synechocystis* sp. PCC 6803, for instance, was adapted to cope with additional (light) energy on account of a higher light harvesting capacity (Dann et al., 2021), auguring well for an effective use of high light intensities. Relatively low growth rates constitute another obstacle for photo-biotechnology (Table 3). Here, the fast growing *Synechococcus elongatus* UTEX 2973 (Ungerer et al., 2018) and the recently described *Synechococcus* sp. PCC 11901 represent a significant progress (Włodarczyk et al., 2020). For the production of fuels or chemicals with (phototrophic) whole-cell factories, solvent tolerance is another desirable trait, for which considerable ALE-based progress recently has been reported for *Synechococcus* (Srivastava et al., 2021).

As mentioned above, phototrophs experience light excess under normal/natural conditions and have evolved multiple mechanisms for excessive energy dissipation (Muramatsu and Hihara, 2012; Canonico et al., 2021). Approaches are needed to avoid such energy loss by overflow valves and to control electron flow. Several studies tackled this aspect in cyanobacteria and green algae. The major protection mechanisms in cyanobacteria are cyclic electron flow around PSI consuming up to 35% of the photosynthetically captured electrons (Theune et al., 2020), flavodiiron proteins, the orange carotenoid protein as main energy dissipater in cyanobacterial antenna systems, and adaptations of the photosynthetic apparatus, e.g., state transitions, antenna sizes, etc. Flavodiiron proteins (Bao et al., 2017) were targeted in several studies (Allahverdiyeva et al., 2015). Knock out strategies increased electron flow and desired product formation (Thiel et al., 2019; Assil-Companioni et al., 2020). As recently reviewed (Bassi and Dall'Osto, 2021; Magdaong and Blankenship, 2018), numerous mechanisms protect green algae from photoinhibition, which should be tackled in a holistic approach.

#### 3.3.3 Biotechnological Application/Engineering of Phototrophs

Several challenges have to be addressed to apply phototrophic organisms for production purposes. Respective processes are often hindered by light limitation, inefficient light utilization, low biomass concentrations, and low production rates. As light supply is a key aspect and a major limiting factor for high-density cultivation of phototrophs, measures are needed to improve light supply and utilization (Stephens et al., 2021). Major factors are the limited absorption capacities and self-shading effects in outdoor applications (Socher et al., 2016). Systems and synthetic biology approaches aim at the engineering of strains with improved photosynthetic performance based on, e.g., improved photosystems, optimal protein allocation, and/or efficient carbon concentration and fixation (Burnap, 2015; Ort et al., 2015; Jahn et al., 2018; Luan et al., 2020; Miao et al., 2020; Roussou et al., 2021). Strategies include the reduction of antenna systems for more efficient light absorption in high-density cultures (Kirst et al., 2014), attenuation of protection mechanisms (Allahverdiyeva et al., 2015; Bassi and Dall'Osto, 2021; Kirilovsky et al., 2014), and streamlining of carbon, energy, and redox metabolism (Veaudor et al., 2020; Roussou et al., 2021; Santos-Merino et al., 2021; Zhou et al., 2021; Jaiswal et al., 2022). Furthermore, operating costs and costs for product removal have to be considered. Substrate and product toxicity constitute a general critical factor for industrial application, which can be mitigated by *in situ* product removal (ISPR) concepts. As an example, a two-liquid-phase system has been demonstrated to stabilize a cyanobacterial CYP450-based process by reducing substrate toxicity and volatility (Hoschek et al., 2019a).

Conventional photobioreactor systems are often limited by low cell densities (Chanquia et al., 2021). Therefore, innovative approaches to overcome light limitation have been developed recently (Johnson et al., 2018; Kirnev et al., 2020; Chanquia et al., 2021), including bioreactors with internal illumination (Heining et al., 2015; Hobisch et al., 2021), biofilm-based concepts (Li et al., 2017), and cell immobilization approaches (Léonard et al., 2010; Polakovič et al., 2017). Biofilm bioreactors enable high surface to volume ratios and thus high biomass concentrations and high light utilization efficiency. Bioreactors with internal light supply enabled a reduced light limitation of suspended cells and a more than two-fold increase in volumetric productivity for photosynthesis-driven 2-methylmaleimide reduction (Hobisch et al., 2021). Further, natural or synthetic consortia recently attracted attention (Hays et al., 2017; Weiss et al., 2017), offering benefits regarding process stability, multistep enzyme cascades, cofactor supply, and light utilization (Fedeson et al., 2021). Hoschek et al. (2019b) demonstrated that a two species biofilm approach enables a process duration of several weeks for cyclohexane hydroxylation at a rate of up to 3.76 g_cyclohexanol_ m^−2^ day^−1^ with reduction equivalents and O_2_ provided by the photosynthetic light reaction in cyanobacteria.

Other critical factors in photobiotechnology, not further addressed here, include temperature and pH control, gas (CO_2_, O_2_) mass transfer, strain-specific nutrient supply, water evaporation, harvesting costs, and sterility. Interested readers are referred to relevant reviews (Fabris et al., 2020; Ullmann and Grimm, 2021; van den Berg et al., 2019; Fernandes et al., 2015).

## 4 Conclusions and Perspectives

Transhydrogenases and NAD^+^ kinases have been shown to be promising targets to tune redox metabolism towards an efficient support of NAD(P)H-dependent productive reactions. The introduction of heterologous NAD(P)H generating enzyme systems into whole-cell biocatalysts on the other hand relieves the dependency of target reactions on the cellular redox metabolism and can be suitable for specific biotransformations. Targeted modifications in central carbon metabolism, such as overexpression and deletion of genes encoding critical enzymes or regulators, can effectively enhance redox-dependent production processes. Respective investigations also provide insight into the intricate regulatory network guiding future pathway and genome-scale engineering efforts to further boost productivities. Whereas the PPP has high potential for the optimization of NADPH availability, EDP, TCA cycle, and respective engineering have been shown to be valuable targets to achieve high NAD(P)H regeneration rates. The TCA-cycle also has high value to drive redox reactions depending on metabolites other than NAD(P)H, e.g., α-KG for oxyfunctionalizations catalyzed by α-KG-dependent dioxygenases.

Cyanobacteria and green algae have great potential as whole-cell redox biocatalysts, making use of water and sunlight to provide reduction equivalents. Recent studies showed that introduction of an artificial electron sink could not only benefit from, but also enhance photosynthetic performance. For high light-to-product efficiencies, efficient electron transport in the photosynthetic apparatus is required. Promising strategies include overexpression of limiting elements, e.g., cytochrome b_6_f, attachment to PSI, and targeted manipulation of protection mechanisms. Further, strategies to avoid intracellular competition are needed, such as attenuating FNR affinity for ferredoxin and thereby making more ferredoxin available for target reactions. Finally, future approaches have to tackle imbalances among redox and energy status of the cells.
